# Unusual Complications in Cocaine Stuffers: A Case Report

**DOI:** 10.5811/cpcem.39681

**Published:** 2025-03-20

**Authors:** Hassan Al-Balushi, Andres Guzman-Soto, Kyle Suen, Al Yaqdhan Al Atbi, Ziad Kazzi, Jonathan de Olano, Todd Taylor

**Affiliations:** * Emory University School of Medicine, Department of Emergency Medicine, Division of Medical Toxicology, Atlanta, Georgia; † Georgia Poison Center, Atlanta, Georgia; ‡ Sohar Hospital, Department of Emergency Medicine, Ministry of Health, Muscat, Oman

**Keywords:** body packing, body stuffing, cocaine, toxicity

## Abstract

**Introduction:**

Body stuffing is defined as ingesting small quantities of drugs in poorly sealed packets, often to avoid repercussions from law enforcement. Cocaine is one of the drugs most commonly involved. Complications reported with stuffing include aspiration, esophageal obstruction, and fatal toxicity. Survival from mechanical airway obstruction due to drug stuffing has not been reported.

**Case Report:**

We present a case of a 32-year-old male who was a cocaine body stuffer, complicated by agitated delirium, cardiotoxicity, and airway obstruction requiring resuscitation, followed by a surgical tracheostomy to retrieve the obstructing cocaine bag. The patient’s hospital course was further complicated by rhabdomyolysis and acute kidney injury requiring dialysis. He was discharged in stable condition after a four-week hospital stay.

**Conclusion:**

This case highlights the severe risks of cocaine body stuffing, including airway obstruction and cocaine-induced arrhythmias. Endotracheal intubation in such cases warrants a careful airway assessment to mitigate the risks of obstructive complications.

## INTRODUCTION

Body stuffing is defined as ingesting small quantities of drugs in poorly sealed packets, often to avoid repercussions from law enforcement.^1^ Cocaine is one of the drugs most commonly involved in stuffing cases. Other drugs include amphetamines and opioids. Complications reported with stuffing include aspiration, esophageal obstruction, and fatal toxicity due to the leakage of the products into the gastrointestinal tract.^2^ Mechanical airway obstruction secondary to drug stuffing is rare, but it carries lethal consequences if not recognized and managed appropriately in a timely manner.

A case of airway obstruction secondary to heroin stuffing was reported by Sullivan et al in 2017.^3^ However, that patient expired in the emergency department (ED) due to asphyxiation from an obstructing bag in the right main bronchus. Prompt identification of airway obstruction by medical personnel may limit further complications in body stuffers. We present a case of a cocaine stuffer who presented with systemic manifestations of cocaine toxicity followed by a mechanical airway obstruction.

## CASE REPORT

**Image 1:**
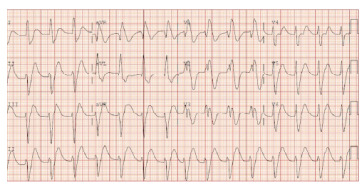
Electrocardiography prior to the administration of sodium bicarbonates in a cocaine body stuffer, demonstrating widening of the QRS-interval.

**Image 2:**
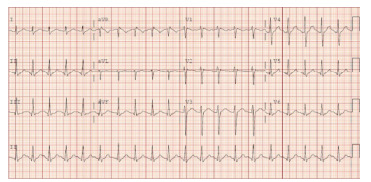
Electrocardiography post-sodium bicarbonate administration in a cocaine body stuffer, demonstrating narrowing of the QRS-interval.

A 32-year-old male was brought in by law enforcement to the ED after reportedly stuffing a suspected illicit drug packet on the street. During initial ED assessment he refused to open his mouth or cooperate. He then had an episode of emesis followed by agitation. Despite administration of midazolam 10 milligrams (mg) and haloperidol 20 mg intramuscularly, he ultimately required intubation via direct laryngoscopy for airway protection and agitated delirium. Once intubated, he was noted to have mydriasis, hypertension, and electrocardiography changes of regular wide complex tachycardia (QRS: 240 milliseconds), right bundle-branch block pattern and a terminal R-wave in AVR (Image 1). he was given a total of 380 milliequivalents of intravenous sodium bicarbonate until the QRS-interval normalized (Image 2). Over the following hours, he was noted to have a high peak airway pressure in the mechanical ventilator and was difficult to ventilate with a bag-valve mask.

**Image 3:**
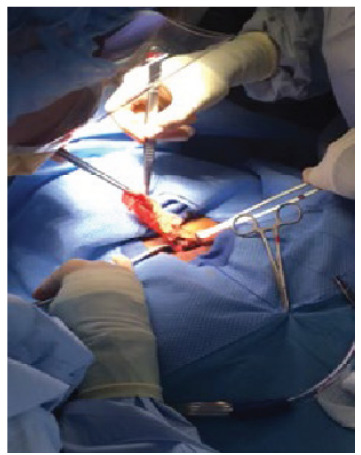
Plastic bag extraction from the trachea in a cocaine body stuffer through tracheostomy.

He was given albuterol, rocuronium, and ketamine without improvement of his airway pressure. Chest radiograph was unremarkable. An emergent bronchoscopy was performed at the bedside that revealed a plastic bag at the distal end of the endotracheal tube. The patient then developed total airway obstruction and cardiac arrest while the physician was attempting to retrieve the bag by bronchoscope. Return of spontaneous circulation was achieved after a left main bronchial stem intubation was performed with standard resuscitation. Emergency tracheostomy was performed in the operating room, and a plastic bag containing a white powder was removed (Image 3). The patient was admitted to the intensive care unit for further stabilization and supportive care. His hospital course was complicated by rhabdomyolysis, elevated liver enzymes, and acute kidney injury requiring hemodialysis. He was discharged in stable condition after four weeks.

## DISCUSSION

We present a case of a cocaine body stuffer who presented with both sympathomimetic toxidrome and airway obstruction. His course was complicated by a cardiac arrest, attributed to possible mechanical airway obstruction. Wide complex tachycardia has a wide range of causes including structural cardiac disease, electrolyte and metabolic derangements, and ischemic heart disease. However, in the context of body stuffing, specifically with cocaine, it should raise the concern about its mechanism as a sodium channel blocker. This clinical effect should be managed aggressively with sodium bicarbonates to avoid further progression to ventricular dysrhythmias and cardiac arrest.

**CPC-EM Capsule**

What do we already know about this clinical entity?

*Body stuffing is the ingestion of poorly sealed drug packets (commonly cocaine) to evade law enforcement, carrying risks of substance toxicity, esophageal obstruction, and airway obstruction*.

What makes this presentation of disease reportable?

*Airway obstruction is a rare complication of body stuffing; this case highlights both cocaine poisoning and mechanical airway obstruction, emphasizing its severity*.

What is the major learning point?

*Physicians should recognize the risk of airway obstruction in suspected body stuffers and take extra precautions during intubation to prevent*

*obstructive complications*.

How might this improve emergency medicine practice?

*It highlights the need for heightened awareness and careful airway management in body stuffing cases, improving patient outcomes and reducing*

*complications*.

Aspiration and subsequent airway obstruction are rare but life-threatening complications of body stuffing that need to be considered. We suspect that our patient aspirated the plastic bag while vomiting when he developed altered mental status and agitation that led to subsequent upper airway obstruction. It is also possible that performance of direct laryngoscopy intubation might have unintentionally pushed the stuffed packet distally in the airway. It has been described in the literature that body stuffers may not actually ingest drug packets but keep them in the oropharynx.^4^

Our case shares similar findings with a report by Narula et al, which described a 23-year-old man who developed airway obstruction and prehospital cardiac arrest after ingesting a small bag containing white powder while attempting to flee from the police. Similar to our case, the diagnosis was made by bronchoscopy after airway obstruction was suspected due to difficulty in bagging the patient, failure to ventilate, and elevated peak pressures on the ventilator.^5^

The risk of toxicity in body stuffing is strongly associated with the type of packaging and the amount of the drug that it contains. An analysis of 683 packages in body stuffers showed that 74% of them used filter paper and 11% single-layer plastic wraps or pouches,^6^ which makes it easy to leak and cause significant toxicity as presented in our case.

## CONCLUSION

We present a case of a cocaine body stuffer who suffered a sympathomimetic toxidrome, cardiac arrest, and airway obstruction by the ingested packet. In addition to benzodiazepines and sodium bicarbonate for the treatment of severe cocaine toxicity, careful consideration should be made when performing endotracheal intubation to assess for foreign bodies and airway obstruction in body stuffers.
